# Rising Cardiovascular Mortality Despite Increased Resource Utilization: Insights From the Nationwide Inpatient Sample Database

**DOI:** 10.7759/cureus.57856

**Published:** 2024-04-08

**Authors:** Chikodili Nebuwa, Omouyi J Omoike, Adeniyi Fagbenro, Fidelis Uwumiro, Efe Erhus, Victory Okpujie, Ifeoluwa Fadeyibi, Onyedikachi Adike, Agatha O Osadolor

**Affiliations:** 1 Internal Medicine, Nuvance Health Medical Practice, New York City, USA; 2 Medicine and Surgery, University of Ottawa, Ottawa, CAN; 3 Internal Medicine, Bowen University College of Health Sciences, Iwo, NGA; 4 Internal Medicine, University of Benin Teaching Hospital, Benin, NGA; 5 Internal Medicine, Central Hospital, Benin, NGA; 6 Internal Medicine, Windsor University School of Medicine, Cayon, KNA; 7 Internal Medicine, Enugu State University Teaching Hospital, Enugu, NGA; 8 Family Medicine, University of Benin Teaching Hospital, Benin, NGA

**Keywords:** nationwide inpatient sample (nis), covid-19, cardiovascular disease, resource utilization, in-hospital mortality

## Abstract

Introduction

The global burden of cardiovascular disease (CVD) has risen over the past decade, potentially escalating resource utilization, morbidity, and mortality. We analyzed trends in hospitalization for CVDs, outcomes of hospitalizations, and the impact of the COVID-19 pandemic on CVD hospitalizations between 2016 and 2020.

Methods

Adult CVD hospitalizations recorded in the 2016-2020 nationwide inpatient sample (NIS) were identified using major diagnostic categories (MDC- class 5). The NIS is the largest all-payer repository of all hospitalizations in the USA within a calendar year. We compared sociodemographic factors and outcomes (mortality, length of stay, and hospital charges) of CVD hospitalization before and during the pandemic using Pearson’s χ2 tests. We used Stata ranking commands and ICD-10 (10th revision of the International Statistical Classification of Diseases and Related Health Problems) codes to identify the most recurring diagnoses associated with CVD mortality during the study period. Trends in mortality and resource use were assessed using the Jonckheere-Terpstra trend test. Hospital charges were adjusted for inflation using the Medical Expenditure Panel Survey index. We used stepwise multivariate logistic regression analyses (P ≤ 0.05 for entry; P > 0.10 for removal) to identify covariates associated with cardiovascular mortality during the study period.

Results

Hospitalizations for CVDs rose from 4,283,502 in 2016 to 4,635,246 in 2019 (P*_trend _*< 0.001) and declined to 3,865,399 in 2020. 452,930 mortalities were recorded during the study period. In-hospital mortality rose from 111,090 (2.6%) in 2016 to 118,825 (2.8%) in 2020 (P*_trend _*< 0.001). Compared with the prepandemic years, mortality rates were higher during the pandemic (108,231 [2.8%] vs. 445,373 [2.5%]; P<0.001), and increased in hospitalizations for hypertensive heart disease with chronic kidney disease (CKD) (15,585 [14.4%] vs. 45,873 [10.3%]; P<0.001), hypertensive heart disease with heart failure (7,468 [6.9%] vs. 21,378 [4.8%]), ventricular tachycardia (2,056 [1.9%] vs. 7,571 [1.7%]; P=0.022), and peripheral angiopathy with gangrene (1,191 [1.1%] vs. 3,118 [0.7%]; P<0.001). CVD hospitalizations totaled 80.3 million hospital days and 39.7 million hospital procedures during the period. The mean number of procedures (3 vs. 2) and mean length of hospital stay (5.6 vs. 4.5 days) increased during the pandemic (P<0.001). The mean hospital cost for CVD increased from US$ 69,394 in 2016 to US$ 89,728 in 2020 (P*_trend _*< 0.001).

Conclusion

CVD mortality increased despite increased resource use over the study period. Hospitalizations during the pandemic had poorer mortality and resource use outcomes than those in the preceding years.

## Introduction

Cardiovascular diseases (CVDs) are the leading cause of global morbidity and mortality, creating significant healthcare and economic challenges [1,2]. The 2016-2020 period has been critical for healthcare systems, especially because of the unprecedented challenges of the COVID-19 pandemic. This timeframe offers a vital opportunity to examine CVD hospitalization trends, outcomes, and resource use, particularly under the influence of the pandemic.

Before 2010, advances in medical technology, pharmacotherapy, and healthcare strategies significantly enhanced the monitoring, diagnosis, and management of CVDs [3-5]. Despite this, the impact of these advancements on CVD hospitalization outcomes has been inconsistent in research findings. The emergence of COVID-19 introduced additional complexities in patient care, affecting not only those infected by the virus but also individuals with preexisting conditions such as CVDs [6,7]. Hospitals face extraordinary challenges, including resource reallocation, changes in admission policies, and heightened risks of virus transmission [8].

While numerous studies have investigated cardiovascular mortality in various CVD patient subgroups, few have concentrated on the overall mortality rates and resource use in hospitalizations from 2016 to 2020. Our study examined how recent medical advancements and pandemic challenges have influenced overall outcomes. Specifically, we evaluated trends in hospitalization, resource use, and in-hospital mortality related to CVD hospitalizations. We also analyzed the most common diagnoses associated with cardiovascular mortality during the study period.

## Materials and methods

Data source

This study used the Nationwide Inpatient Sample (NIS) from January 1, 2016, to December 2020 to analyze adult hospitalizations for CVDs and disorders. The NIS, part of the Healthcare Cost and Utilization Project (HCUP), is managed by the Agency for Healthcare Research and Quality (AHRQ) and is the largest inpatient claims-based database in the USA. It covers over 7 million unweighted hospital stays annually and over 35 million hospitalizations when weighted. The database includes data from various hospital types across urban and rural areas, representing up to 97% of US hospitalizations [9,10]. Since 2016, the NIS has consistently used the International Classification of Diseases, Tenth Revision (ICD-10-CM/PCS) for coding diagnoses and procedures. The NIS records detailed information on patient demographics, diagnoses, procedures, duration of hospital stay, care costs, and outcomes, making it ideal for trend analysis across years. It records all-cause mortality, length of hospital stay (LOS), and total hospital costs (THC) in US dollars. This study complied with the Strengthening the Reporting of Observational Studies in Epidemiology reporting guidelines and the study design checklist by Khera et al. (2017) [7,11].

Study selection and endpoints

**Figure 1:**
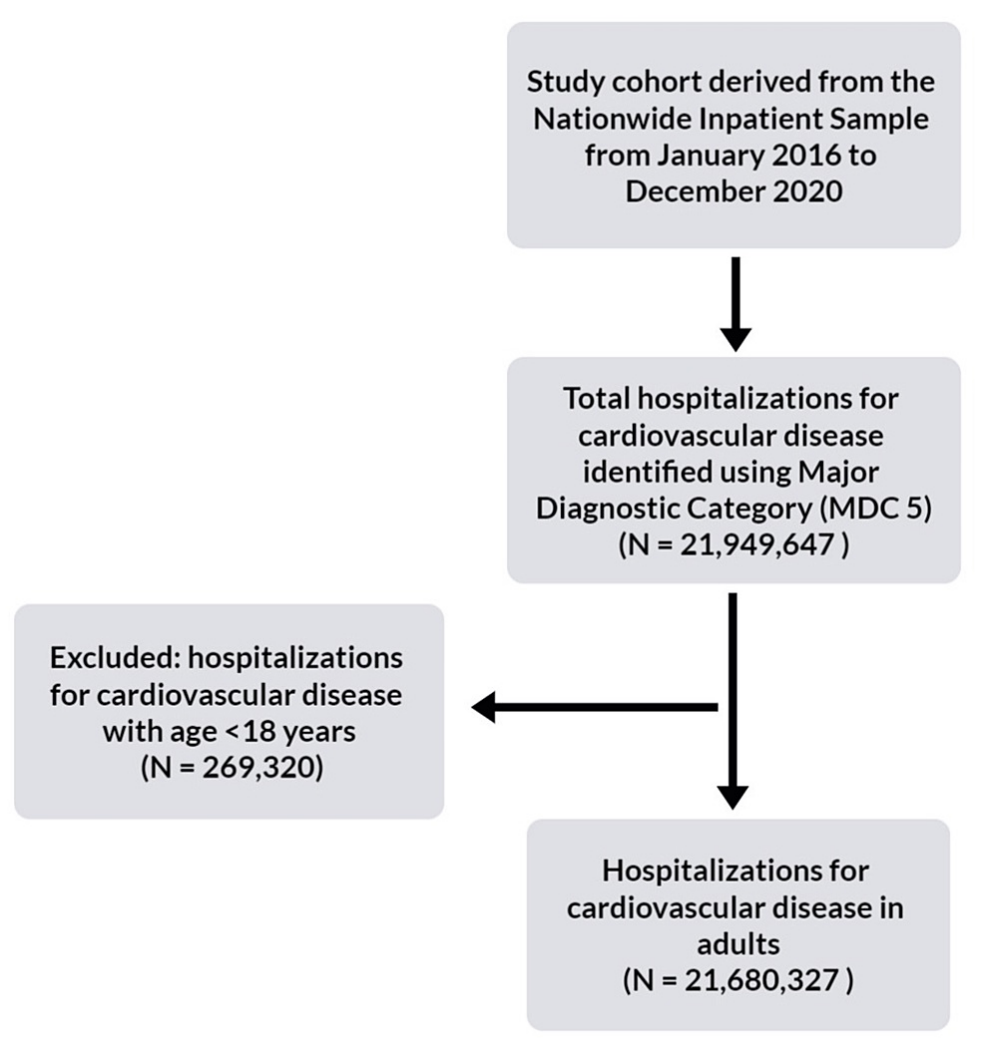
Study inclusion and exclusion MDC-5: Major diagnostic category class 5: Diseases and disorders of the circulatory system

We identified adult CVD hospitalizations using major diagnostic categories (MDC-5), analyzing trends in hospitalization, all-cause mortality, and prevalent mortality-related diagnoses. In addition, we compared hospitalization rates, patient profiles, and mortality between pre- and post-pandemic years, along with trends in hospital costs and length of stay. The process of derivation of our study cohort is summarized in Figure 1.

Statistical analysis

Initially, we categorized all CVD hospitalizations into two periods: prepandemic (2016-2019) and pandemic (2020). This categorization was performed to allow a comparison of sociodemographic characteristics and mortality rates between prepandemic and pandemic years, to investigate the potential impacts of the pandemic, and to address any issues of secular confounding. We used Pearson’s χ2 analysis or Fisher’s exact test to compare categorical variables. For continuous variables, we used t-tests and the Wilcoxon rank-sum test to analyze data with normal and nonnormal distributions. The normality of numerical data was assessed using the Kolmogorov-Smirnov test. Because of the large sample size and the increased likelihood of finding low p-values by chance, p-values of <0.05 were considered statistically significant [12,13]. Following this, we analyzed outcome trends within the overall CVD cohort and specifically between the prepandemic and pandemic years. Survey analysis and ranking commands were used to identify the commonest principal diagnoses associated with cardiovascular mortality and the Jonckheere-Terpstra trend tests were used to assess trends in hospital costs and mortality. Hospitalization costs were adjusted for inflation to reflect the 2020 $US using the Medical Expenditure Panel Survey index. All statistical analyses were performed using Stata software version 7MP (StataCorp LLC, College Station, TX, USA). We used stepwise multivariable logistic regression analyses to identify covariates associated with cardiovascular mortality during the study period (P ≤ 0.05 for entry; P > 0.10 for removal). Missing covariate data were assessed using the “mdesc” Stata commands, and missing numerical data were treated using multiple mean imputation. Multicollinearity was assessed by computing the variance inflation factor (VIF). Covariates with VIF values >5 were excluded from the regression analysis to maintain a smaller and more accurate regression model.

## Results

Between January 1st, 2016, and December 31st, 2020, approximately 21,680,327 hospitalizations for CVDs were recorded. In 2020, there were 3,865,399 hospitalizations for cardiovascular disorders. Compared with prepandemic hospitalizations, hospitalizations during the pandemic had higher Charlson Comorbidity Index (CCI) scores (CCI ≥ 3: 2,118,239 [54.8%] vs. 931,687 [52.3%], P < 0.001), a higher proportion of older adults (2,848,799 [73.7%] vs. 12,951,453 [72.7%]; P < 0.001), more females (P < 0.001), fewer weekend admissions (P = 0.021) and higher hospitalizations at urban teaching hospitals (P < 0.001; Table 1). There were more hospitalizations for CVDs in the first quarter of 2020 compared with the first quarter admissions in the prepandemic years (P < 0.001). Hospitalizations in 2020 had higher prevalence of previous myocardial infarction (1,186,677 [30.7%] vs. 5,130,699 [28.8%]; P < 0.001), congestive heart failure (2,187,816 [56.6%] vs. 9,335,022 [52.4%]; P < 0.001), cerebrovascular disease (258,982 [6.7%] vs. 1,104,526 [6.2%]; P < 0.001), obesity (413,598 [10.7%] vs. 2,191,236 [12.3%]; P < 0.001), smoking (1,677,583 [43.4%] vs. 7,963,272 [44.7%]; P = 0.0001), dyslipidemia (1,855,392 [48%] vs. 9,049,983 [50.8%]; P < 0.001), renal disease (1,364,486 [35.3%] vs. 5,736,409 [32.2%]; P < 0.001), complicated diabetes (1,024,331 [26.5%] vs. 3,562,986 [20.0%]; P < 0.001), rheumatoid disease (119,827 [3.1%] vs. 534,448 [3.0%]; P < 0.001), liver disease (173,943 [4.5%] vs. 641,337 [3.6%]; P < 0.001), and cancer (193,270 [5.0%] vs. 801,672 [4.5%]; P < 0.001), whereas prevalence of dementia (1,140,155 [6.4%] vs. 235,789 [6.1%]; P < 0.001), uncomplicated diabetes (3,598,615 [20.2%] vs. 595,271 [15.4%]; P < 0.001), and hypertension (1,140,155 [6.4%] vs. 235,789 [6.1%]; P < 0.001) were higher in the prepandemic hospitalizations (Table 1).

**Table 1:** Baseline characteristics of adults hospitalized for cardiovascular disease: prepandemic (2016–2019) and pandemic (2020) hospitalizations Numerical data are presented as mean ± SD; categorical data are presented as absolute numbers (N) and proportion (%). LOD: likelihood of dying; DRG: diagnosis-related groups; LOF: loss of function; LOS: length of hospital stay; MI: myocardial infarction; CHF: congestive heart failure; SNF: skilled nursing facility; US$: United States Dollar; SD: standard deviation; COPD: chronic obstructive pulmonary disease ^a^Sundararajan's adaptation of the modified Deyo's CCI, which offers a refined approach for population-based investigations. This adaptation classifies the CCI into four distinct groups, each indicative of escalating mortality risk. A CCI score surpassing 3 is associated with an approximate 25% 10-year mortality rate, whereas scores of 2 or 1 correspond to 10% and 4% 10-year mortality rates, respectively.

Patient and Hospital Variables	Prepandemic Hospitalizations (N=17,814,928)	Pandemic Hospitalizations (N=3,865,399)	P-Value
Female sex, n(%)	9,869,470 (55.4)	2,180,085 (56.4)	<0.001
Mean age (SD)	67.9 (7.5)	68 (7.6)	0.348
Age Categories, n(%)			<0.001
18 – 40 years	694,782 (3.9)	154,616 (4.0)	
41 – 60 years	570,078 (3.2)	85,039 (2.2)	
61 – 80 years	8,551,165 (48.0)	1,936,565 (50.1)	
> 80 years	4,364,657 (24.5)	912,234 (23.6)	
Race and Ethnicity, n(%)			0.890
White	12,238,256 (68.7)	2,678,722 (69.3)	
Black	2,939,463 (16.5)	645,522 (16.7)	
Hispanic	1,638,973 (9.2)	328,559 (8.5)	
Asian or Pacific Islander	427,558 (2.4)	88,904 (2.3)	
Native American	89,075 (0.5)	23,192 (0.6)	
Other	481,003 (2.7)	100,500 (2.6)	
Charlson Comorbidity Index Scores^a^, n(%)			<0.001
0	1,852,753 (10.4)	351,751 (9.1)	
1	3,295,762 (18.5)	684,176 (17.7)	
2	3,349,206 (18.8)	711,233 (18.4)	
≥ 3	9,317,207 (52.3)	2,188,239 (54.8)	
Median Annual Income in the Patient’s Zip Code, n(%)			0.466
$1–$45,999	5,522,628 (31.0)	1,225,331 (31.7)	
$46,000 – $58,999	4,899,105 (27.5)	1,070,715 (27.7)	
$59,000 – $78,999	4,150,878 (23.3)	865,849 (22.4)	
≥ $79,000	3,260,132 (18.3)	703,503 (18.2)	
All Patient-Refined-DRG: Risk of Mortality, n(%)			<0.001
Minor LOD	3,848,024 (21.6)	889,042 (23.0)	
Moderate LOD	5,754,222 (32.3)	1,171,216 (30.3)	
Major LOD	5,878,926 (33.0)	1,236,928 (32.0)	
Extreme LOD	2,351,571 (13.2)	568,214 (14.7)	
All Patient-Refined-DRG: Severity of Illness, n(%)			<0.001
Minor LOF	2,315,941 (13.0)	715,099 (18.5)	
Moderate LOF	6,074,890 (34.1)	1,495,909 (38.7)	
Major LOF	5,504,813 (30.9)	1,175,081 (30.4)	
Extreme LOF	3,010,723 (16.9)	479,309 (12.4)	
Insurance Type, n(%)			0.004
Medicare	11,900,372 (66.8)	2,539,567 (65.7)	
Medicaid	1,906,197 (10.7)	432,925 (11.2)	
Private	3,384,836 (19.0)	753,753 (19.5)	
Uninsured	623,522 (3.5)	139,154 (3.6)	
Hospital Region, n(%)			0.999
Northeast	3,349,206 (18.8)	711,233 (18.4)	
Midwest	4,008,359 (22.5)	858,119 (22.2)	
South	732,193,541 (41.1)	1,619,602 (41.9)	
West	3,135,427 (17.6)	680,310 (17.6)	
Hospital Bed Size, n(%)			0.082
Small	3,420,466 (19.2)	784,676 (20.6)	
Medium	5,255,404 (29.5)	1,097,773 (28.4)	
Large	9,139,058 (51.3)	1,971,353 (51.0)	
Hospital Ownership, n(%)			0.993
Government, nonfederal	1,781,493 (10.0)	386,540 (10.0)	
Private, non-profit	13,236,492 (74.3)	2,891,318 (74.8)	
Private, investor-own	2,796,944 (15.7)	587,541 (15.2)	
Hospital Location and Teaching Status, n(%)			<0.001
Rural	1,389,564 (7.8)	297,636 (7.7)	
Urban nonteaching	3,669,875 (20.6)	668,714 (17.6)	
Urban teaching	12,737,674 (71.5)	2,887,453 (74.7)	
Weekend admission, n(%)	3,794,580 (21.3)	800,138 (20.7)	0.021
Mean LOS, days	4.5	5.6	<0.001
Mean number of procedures, days	2.1	2.4	<0.001
Elective admissions, n(%)	2,405,015 (13.5)	514,098 (13.6)	0.211
Disposition of the Patient ( Discharge Status), n(%)			<0.001
Routine home discharge	10,688,957 (60.0)	2,296,047 (59.4)	
Transfer to a short-term hospital	623,522 (3.5)	123,693 (3.2)	
Other transfers (SNF, intermediate care facility)	2,636,609 (14.8)	483,175 (12.5)	
Home healthcare	3,135,427 (17.6)	776,945 (20.1)	
Against medical advice	285,039 (1.6)	77,308 (2.0)	
Died in the hospital	463,188 (2.6)	108,231 (2.8)	
Discharge Quarter, n(%)			<0.0001
First	4,542,807 (25.5)	1,101,639 (28.5)	
Second	4,489,362 (25.2)	819,464 (21.2)	
Third	4,329,028 (24.3)	966,350 (25.0)	
Fourth	4,453,732 (25.0)	974,081 (25.2)	
Comorbidities, n(%)			
Old MI	5,130,699 (28.8)	1,186,677 (30.7)	<0.0001
CHF	9,335,022 (52.4)	2,187,815 (56.6)	<0.0001
Peripheral vascular disease	3,402,651 (19.1)	726,695 (18.8)	0.112
Cerebrovascular disease	1,104,525 (6.2)	258,982 (6.7)	<0.0001
Dementia	1,140,155 (6.4)	235,789 (6.1)	<0.0001
Hypertension	6,217,410 (34.9)	1,039,792 (26.9)	<0.0001
Dyslipidemia	8,551,165 (48.0)	1,963,623 (50.8)	<0.0001
Obesity	1,906,197 (10.7)	475,444 (12.3)	<0.0001
Smoking	7,731,679 (43.4)	1,727,833 (44.7)	0.0001
Renal disease	5,736,407 (32.2)	1,364,486 (35.3)	<0.0001
Uncomplicated diabetes types 1 and 2	3,598,615 (20.2)	595,271 (15.4)	<0.0001
Complicated diabetes types 1 and 2	3,562,986 (20.0)	1,024,331 (26.5)	<0.0001
COPD	4,988,180 (28.0)	1,090,041 (28.2)	0.294
Rheumatoid disease	534,448 (3.0)	119,827 (3.1)	0.001
Peptic ulcer disease	142,519 (0.8)	30,923 (0.8)	0.582
Liver disease	641,337 (3.6)	173,943 (4.5)	<0.0001
Cancer	801,672 (4.5)	193,270 (5.0)	<0.0001
Hemiplegia or paraplegia	124,704 (0.7)	30,923 (0.8)	<0.0001
AIDS	35,630 (0.2)	7,730 (0.2)	0.224

Trends in hospitalization rates and mortality

**Figure 2:**
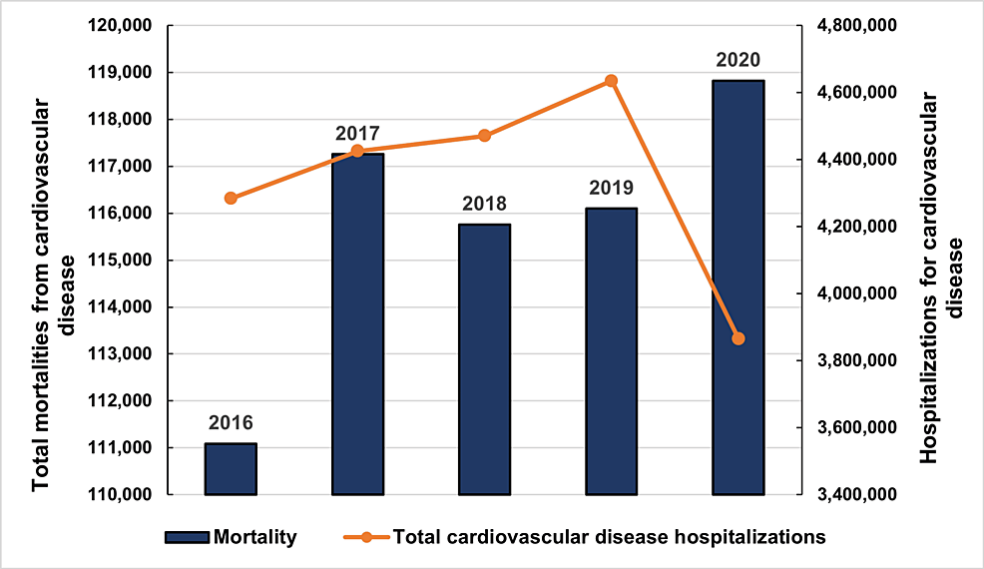
Annual trends in hospitalization and mortality due to cardiovascular disease over the study period Data is presented as total numbers

Hospitalization for CVD rose from 4,283,502 in 2016 to 4,635,246 in 2019 (P<0.001) and declined to 3,865,399 in 2020 (Figure 2). A total of 452,930 deaths were recorded during the study period. All-cause in-hospital mortality rose from 111,090 (2.6%) in 2016 to 118,825 (2.8%) in 2020 (P<0.001) despite fewer hospitalizations in 2020 (Figure 2).

Compared with prepandemic years, mortality rates were higher during the pandemic (108,231 [2.8%] vs. 445,373 [2.5%]; P < 0.001; Table 2). Mortality was observed to have increased by 1.1% (42,519) in men, 0.7% (27,058) in Black Americans, and 1% (38,654) in patients with a higher CCI score of ≥ 3 (Table 2). The greatest increase in mortality rates was observed among individuals aged 61-80 years (P < 0.001), whereas mortality declined by 4.5% (173,943) among individuals older than 80 years.

**Table 2:** All-cause in-hospital mortality stratified by sex, age, race, mean household income, and comorbidity burden Data is presented as total numbers (percentages). P-values are significant at values <0.05. *Stratified into four quartiles based on median annual income in the patient’s zip code: the 0–25th percentile (first quartile, $1–$45,999), the 26th–50th percentile (second quartile, $46,000–$58,999), the 51st–75th percentile (third quartile, $59,000–$78,999), and the 76th–100th percentile (fourth quartile, $≥79,000).

Variable	Prepandemic, (N=17,814,928)	Pandemic, (N=3,865,399)	P-value
Total mortality	445,373 (2.5)	108,231 (2.8)	<0.001
Gender			0.008
Female	198,191 (44.5)	46,972 (43.4)	
Male	247,182 (55.5)	61,259 (56.6)	
Race and Ethnicity			0.579
White	319,778 (71.8)	77,385 (71.5)	
Black Americans	61,016 (13.7)	15,585 (14.4)	
Hispanics	35,630 (8.0)	8,334 (7.7)	
Median Household Income (Quartiles)*			0.528
0–25th	132,276 (29.7)	33,876 (31.3)	
26–50th	124,259 (27.9)	30,305 (28.0)	
56 -75^th^	105,108 (23.6)	24,352 (22.5)	
76–100th	83,285 (18.7)	19,806 (18.3)	
Charlson Comorbidity Index			<0.001
0	15,588 (3.5)	3,788 (3.5)	
1	52,554 (11.8)	12,122 (11.2)	
2	69,478 (15.6)	16,451 (15.2)	
≥3	308,198 (69.2)	75,978 (70.2)	
Age Categories			<0.001
18 – 40	10,244 (2.3)	2,814 (2.6)	
41 - 60	61,016 (13.7)	15,909 (14.7)	
61 – 80	205,317 (46.1)	53,358 (49.3)	
> 80	168,796 (37.9)	36,149 (33.4)	

**Figure 3:**
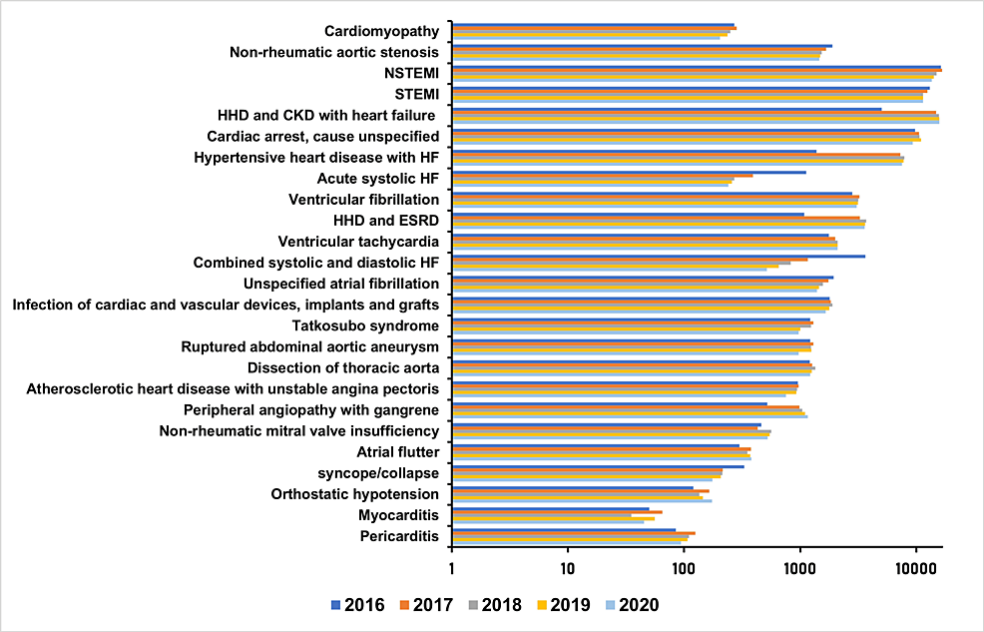
Annual trends for the most common diagnoses associated with mortality during the study period NSTEMI: non–ST‐segment–elevation myocardial infarction; STEMI: ST-segment–elevation myocardial infarction; HHD: home hemodialysis; ESRD: end-stage renal disease; HF: heart failure Data is presented in the logarithmic scale

We identified the most frequently occurring primary diagnoses associated with CVD mortality. Figure 3 depicts the trend of the top 25 principal diagnoses associated with mortality during the study period. The leading causes of mortality included myocardial infarction, hypertensive heart disease and heart failure with or without coexisting chronic kidney disease, unspecified cardiac arrest, atrial fibrillation, nonrheumatic aortic stenosis, and ventricular arrhythmias.

Mortality from hypertensive heart disease and heart failure with coexisting chronic kidney disease, hypertensive heart disease with heart failure, ventricular arrhythmias, peripheral angiopathy with gangrene, nonrheumatic mitral valve insufficiency, atrial flutter, orthostatic hypotension, and pericarditis increased over the study period. Conversely, mortality from cardiomyopathies, nonrheumatic aortic stenosis, acute systolic heart failure, combined systolic and diastolic heart failure, and syncope decreased over the same period.

Table 3 compares mortality rates for the top 25 causes of CVD mortality between the prepandemic and pandemic years. Notable increases were observed in mortality due to conditions such as hypertensive heart disease with chronic kidney disease and heart failure (15,585 [14.4%] vs. 45,873 [10.7%]; P < 0.001), hypertensive heart disease with heart failure (7,468 [6.9%] vs. 21,378 [4.8%]; P < 0.001), ventricular tachycardia (2,056 [1.9%] vs. 7,571 [1.7%]; P = 0.022), and peripheral angiopathy with gangrene (1,191 [1.1%] vs. 3,118 [0.7%]; P < 0.001). In contrast, significant reductions were noted in hospitalizations for non-ST‐segment-elevation myocardial infarction (NSTEMI) (61,907 [13.9%] vs. 13,529 [12.5%]; P < 0.001), systolic heart failure (14,697 [3.6%] vs. 1,407 [1.3%]; P < 0.001), unspecified atrial fibrillation (6,681 [1.5%] vs. 1,408 [1.3%]; P = 0.013), and ruptured abdominal aortic aneurysms (4,891 [1.1%] vs. 976 [0.9%]; P = 0.014).

**Table 3:** Most common primary diagnoses associated with cardiovascular disease mortality: prepandemic vs. pandemic hospitalizations Data has been presented as total mortality for each diagnosis (N) and proportion of total mortalities (%). NSTEMI: non-ST-elevation myocardial infarction; STEMI: ST-elevation myocardial infarction; HHD: hypertensive heart disease; CKD: chronic kidney disease; HF: heart failure; ESRD: end-stage renal disease; AAA: abdominal aortic aneurysm

Diagnoses	Prepandemic, N(%)	Pandemic, N(%)	P-value
Total mortality	445,373 (2.5)	108,231 (2.8)	<0.001
NSTEMI	61,907 (13.9)	13,529 (12.5)	<0.001
STEMI	47,655 (10.7)	11,364 (10.5)	0.364
HHD and CKD associated with heart failure	45,873 (10.3)	15,585 (14.4)	<0.001
Cardiac arrest, cause unknown	40,084 (9.0)	9,200 (8.5)	0.056
Hypertensive heart disease with HF	21,378 (4.8)	7,468 (6.9)	<0.001
Systolic HF	14,697 (3.6)	1,407 (1.3)	<0.001
Ventricular fibrillation	12,025 (2.7)	3,030 (2.8)	0.316
HHD and ESRD	10,244 (2.3)	3,572 (3.3)	<0.001
Ventricular tachycardia	7,571 (1.7)	2,056 (1.9)	0.022
Combined systolic (congestive) and diastolic (congestive) HF	7,125 (1.6)	541 (0.5)	<0.001
Infection and inflammatory reaction due to cardiac and vascular devices, implants, and grafts	7,126 (1.6)	1,623 (1.5)	0.417
Unspecified atrial fibrillation	6,681 (1.5)	1,407 (1.3)	0.013
Nonrheumatic aortic (valve) stenosis	6,681 (1.5)	1,408 (1.3)	0.193
Takotsubo syndrome	4,899 (1.1)	974 (0.9)	0.013
AAA ruptured	4,898(1.1)	976 (0.9)	0.014
Dissection of the thoracic aorta	4,899 (1.1)	1,191 (1.1)	0.943
Atherosclerotic heart disease of native coronary artery with unstable angina pectoris	3,561 (0.8)	758 (0.7)	0.067
Peripheral angiopathy with gangrene	3,118 (0.7)	1,191 (1.1)	<0.001
Nonrheumatic mitral valve insufficiency	1,781 (0.4)	541 (0.5)	0.250
Atrial flutter	1,336 (0.3)	433 (0.4)	0.241
Syncope and collapse	891 (0.2)	216 (0.2)	0.097
Orthostatic hypotension	445 (0.1)	217 (0.2)	0.167

On multivariable regression analysis, older age, Asian/Pacific Islander or Native American race, weekend hospitalization, hospitalizations for individuals at extreme likelihood of dying or with extreme loss of function, and other noncardiac comorbidities such as dementia, chronic liver disease, hemiplegia or paraplegia, cancer, and COVID-19 were associated with a greater likelihood of mortality during the study period (Table 4).

**Table 4:** Multivariable logistic regression of factors associated with cardiovascular mortality CI: confidence interval; DRG: diagnosis related groups; LOF: loss of function; LOD: likelihood of mortality; COPD: chronic obstructive pulmonary disease. Reference = subclass to which other subclasses are compared in the specific category during multivariable logistic regression.

Mortality	Odds Ratio	Standard Error	P-Value	95% CI
Race				
White	Reference	Reference	Reference	Reference
Black	0.980	0.012	0.085	0.956–1.003
Hispanic	0.961	0.016	0.013	0.9318–0.992
Asian/Pacific Islander	1.067	0.025	0.005	1.019–1.117
Native American	1.167	0.057	0.002	1.059–1.285
Other races	1.128	0.0268	<0.001	1.077–1.182
Age	1.020	0.001	<0.001	1.018–1.021
Age Categories				
18 to 40	Reference	Reference	Reference	Reference
41 to 60	0.820	0.024	<0.001	0.772–0.867
61 to 80	0.729	0.028	<0.001	0.676–0.785
>80	0.782	0.037	<0.001	0.713–0.858
Charlson Comorbidity Index				
0	Reference	Reference	Reference	Reference
1	0.972	0.022	0.223	0.930–1.017
2	0.835	0.020	<0.001	0.797–0.875
3	0.801	0.021	<0.001	0.761–0.843
Weekend vs. Weekday admission	1.029	0.009	0.001	1.012–1.048
Elective vs. Emergency admission	0.914	0.017	<0.001	0.882–0.947
All Patient Refined-DRG Risk of Mortality Subclass				
No LOD	Reference	Reference	Reference	Reference
Minor LOD	0.015	0.001	<0.001	0.013–0.016
Moderate LOD	0.050	0.001	<0.001	0.048–0.053
Major LOD	0.198	0.002	<0.001	0.193–0.202
Extreme LOD	1.430	0.002	<0.001	1.035 -2.024
All Patient Refined-DRG Illness Severity Subclass				
No LOF	Reference	Reference	Reference	Reference
Minor LOF	0.232	0.010	<0.001	0.213–0.252
Moderate LOF	0.207	0.004	<0.001	0.199–0.216
Major LOF	0.336	0.004	<0.001	0.329–0.343
Extreme LOF	1.13	0.003	<0.001	1.01–1.46
Comorbidities				
Cerebrovascular Disease	1.003	0.013	0.820	0.978–1.029
Dementia	1.100	0.015	<0.001	1.071–1.129
COPD	0.803	0.007	<0.001	0.789–0.817
Rheumatoid disease	0.900	0.021	<0.001	0.858–0.938
Peptic ulcer disease	0.931	0.031	0.031	0.872–0.993
Mild liver disease	1.100	0.022	<0.001	1.057–1.144
Moderate/severe liver disease	1.965	0.052	<0.001	1.867–2.069
Uncomplicated Diabetes	0.949	0.011	<0.001	0.928–0.971
Complicated diabetes	0.813	0.009	<0.001	0.797–0.830
Hemiplegia/paraplegia	1.031	0.029	0.286	0.975–1.090
Chronic renal disease	0.991	0.010	0.409	0.971–1.012
Cancer	1.064	0.019	0.001	1.027–1.101
Metastatic cancer	1.439	0.034	<0.001	1.374–1.507
AIDS	0.986	0.077	0.860	0.847–1.148
COVID-19	1.220	0.060	<0.001	1.107–1.344

Resource use trends

Hospitalizations for CVD between 2016 and 2020 totaled 80.3 million hospital days and 39.7 million hospital procedures. Compared with the prepandemic years, there was an increase in the mean number of procedures (3 vs. 2 procedures; P < 0.001) and mean LOS during the pandemic (5.6 vs. 4.5 days; P < 0.001). Significant increases in the LOS were recorded among females, males, patients with CCI ≥ 2, Black Americans, Asians/Pacific Islanders, and Native Americans (Table 5).

**Table 5:** Mean hospital stay between prepandemic and pandemic hospitalizations All values are mean hospital stay presented in days.

Variable	Prepandemic Hospitalizations	Pandemic Hospitalizations	P-Value
Overall	4.5	5.6	<0.001
Gender			<0.001
Female	4.0	5.0	
Male	4.7	4.9	
Race and Ethnicity			<0.001
White	4.6	4.7	
Black Americans	5.1	5.3	
Hispanics	4.7	4.8	
Asian or Pacific Islanders	4.7	5.0	
Native Americans	4.7	5.3	
Others	4.8	4.9	
Median Household Income (Quartiles)			0.236
0–25th	4.8	5.0	
26–50th	4.6	4.8	
56 -75^th^	4.6	4.7	
76–100th	4.6	4.7	
Charlson Comorbidity Index			<0.001
0	2.6	2.9	
1	3.6	3.6	
2	4.1	4.3	
≥3	5.2	6.0	
Age Categories			0.108
18 - 40	4.7	2.9	
41 - 60	4.4	3.6	
61 - 80	4.9	4.9	
> 80	4.6	4.6	
Hospital Location And Teaching Status			0.034
Rural	3.7	3.9	
Urban nonteaching	4.1	4.2	
Urban teaching	4.9	5.1	
Hospital region			0.256
Northeast	4.9	5.0	
Midwest	4.6	4.7	
South	4.8	4.9	
West	4.4	4.5	

**Figure 4:**
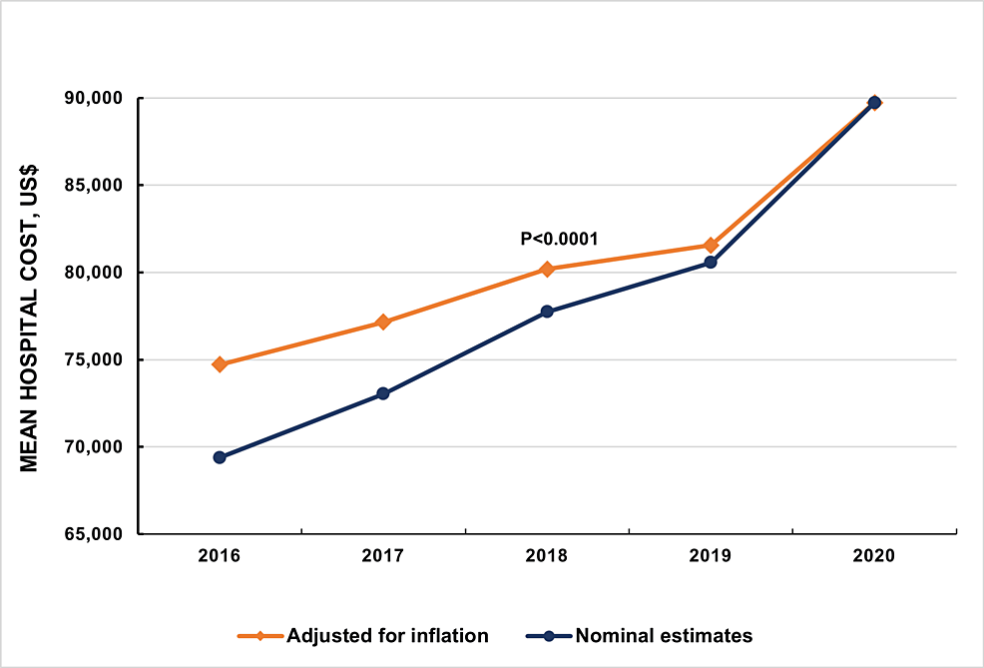
Trends in the mean hospital cost for cardiovascular disease care Nominal estimates represent unadjusted mean hospital costs.

After adjusting for inflation, the mean hospital cost for CVD care was observed to have increased from US$69,394 in 2016 to US$89,728 in 2020 (P < 0.001; Figure 4).

## Discussion

In our study, we observed a notable increase in CVD mortality, especially among males, Black Americans, and those with multiple comorbidities. Historical data from 1969 to 2003 highlighted escalating racial disparities in cardiovascular mortality in the United States, with Black individuals experiencing significantly higher rates than other races [14]. Recent empirical data points toward a disconcerting development: the previously documented reduction in cardiovascular mortality rates, observed until 2010, started a period of stagnation after 2010. This was evident in heart disease, stroke, diabetes, and hypertension, with the most pronounced disparities observed among Black individuals [15]. The index study identified the principal diagnoses contributing to CVD mortality within the examined timeframe. It identified myocardial infarction, hypertensive heart disease occurring with chronic kidney disease, unspecified cardiac arrest, and hypertensive heart disease complicated by heart failure as the foremost causes of mortality. Historically, ischemic heart disease and congestive heart failure have been major contributors to CVD mortality in the United States [16]. These findings elucidate the persistent influence of recognized conditions on CVD mortality, necessitating a thorough reevaluation of current screening and management guidelines for these diseases to improve case finding and early treatment. Additionally, the index findings indicate that the COVID-19 pandemic significantly impacted CVD mortality, with a paradoxical increase in deaths despite decreased hospitalizations due to pandemic-related restrictions [17], highlighting the pandemic’s impact on CVD patients.

During the pandemic, inpatient mortality from ischemic heart disease and cardiac arrests decreased, likely due to people avoiding hospital visits for fear of COVID-19 exposure, affecting the number of cardiac-related deaths recorded in this setting. Hospitals’ reallocation of resources to COVID-19 care may also have limited their capacity to treat other conditions, including ischemic heart disease [18]. In addition, lifestyle changes due to lockdowns and social distancing may reduce the occurrence of cardiac events. However, overall mortality from ischemic heart disease reportedly increased across all hospital settings during the pandemic [19]. Importantly, there was a significant increase in mortality among individuals with both cardiovascular and renal comorbidities, which was likely exacerbated by the combined impact of these conditions and COVID-19 [20].

Before 2010, advancements in medical technology, improved healthcare policies, and increased public awareness contributed to a significant reduction in CVD mortality, aided by better management of risk factors and the introduction of effective treatments. However, after 2010, CVD mortality rates plateaued and then increased because of factors such as an aging population and rising obesity and diabetes rates, indicating a need for newer, more targeted strategies to address the evolving nature of CVD [21].

Central to the findings of the index study is the increased mortality observed in conditions such as hypertensive heart disease and heart failure, particularly when coexisting with chronic kidney disease. This observed increase reiterates the complex impact of hypertension and chronic kidney disease on cardiovascular health, where these conditions mutually exacerbate one another, presenting significant challenges to existing treatment protocols [22]. There is a need to continue strengthening care approaches that simultaneously address these comorbidities or prevent them altogether while rapidly adopting newer treatments proven to improve disease progression and outcomes. A recent approach to managing hypertension in patients with CKD involves a comprehensive strategy that emphasizes accurate blood pressure measurement as the essential first step. Dietary sodium restriction is recommended to improve blood pressure control, particularly in patients treated with renin-angiotensin system blockers [23]. The first-choice antihypertensive agents for patients with high albuminuria are angiotensin-converting enzyme inhibitors or angiotensin receptor blockers (ARBs) [24]. Long-acting dihydropyridine calcium channel blockers and diuretics are considered reasonable second and third-line options. For treatment-resistant hypertension, the addition of spironolactone is advised, although the risk of hyperkalemia limits its use in moderate to advanced CKD [25]. The Chlorthalidone in Chronic Kidney Disease (CLICK) trial highlighted the effectiveness of thiazide-like diuretics for patients with stage IV CKD and uncontrolled hypertension, including those resistant to treatment. Chlorthalidone can also reduce the risk of hyperkalemia, allowing the concurrent use of spironolactone; however, this combination requires careful monitoring. Recent advancements in blood pressure management have resulted in the development of novel agents. These include the nonsteroidal mineralocorticoid receptor antagonist ocedurenone, the aldosterone synthase inhibitor baxdrostat, and the dual endothelin receptor antagonist aprocitentan. These treatments show promise for enhanced effectiveness in blood pressure control. The KARDIA-1 study, a phase 2 clinical trial, has revealed promising results for zilebesiran, an experimental antihypertensive drug developed by Alnylam Pharmaceuticals. In adults with mild to moderate hypertension, a single injection of zilebesiran was found to effectively reduce blood pressure for up to six months. In addition, the drug demonstrated a favorable profile regarding side effects, indicating that it could be a well-tolerated option for hypertension management. This finding indicates that zilebesiran may offer a convenient and long-lasting treatment alternative for individuals with high blood pressure [26].

The increase in mortality associated with peripheral angiopathy with gangrene and ventricular arrhythmias could be indicative of late-stage disease presentations or reflect the challenges of managing these complex conditions. Studies have found that even asymptomatic peripheral arterial disease is associated with increased CVD mortality [27]. Peripheral angiopathy with gangrene, primarily a consequence of advanced peripheral artery disease, often coexists with diabetes and other cardiovascular risk factors and requires aggressive management, including strict glycemic control, lipid management, and hypertension treatment. Other studies have similarly found an increase in mortality due to ventricular arrhythmias [28]. Advancements in diagnostic technology have led to the efficient detection and reporting of ventricular arrhythmias, thereby influencing mortality statistics. Additionally, lifestyle factors such as rising obesity rates, physical inactivity, and the prevalence of other cardiovascular risk factors could have played a significant role.

The current study also revealed a significant decline in mortality from cardiomyopathies, nonrheumatic aortic stenosis, and acute systolic heart failure. This positive trend is likely due to advancements in diagnostic techniques, refinement of treatment protocols, and implementation of enhanced patient management strategies developed recently. In addition, the decrease in mortality rates for syncope and combined systolic and diastolic heart failure further exemplifies the beneficial impact of these medical advancements. Despite these improvements, these conditions continue to be among the top 20 most common causes of cardiovascular mortality in the United States.

The COVID-19 pandemic introduced additional complexity to this landscape. Notably, there was an increase in hospitalizations for conditions such as hypertensive heart disease with chronic kidney disease and heart failure. This trend could be a consequence of the added strain on healthcare systems, delayed or altered treatment protocols for non-COVID conditions, and the direct and indirect impacts of the COVID-19 virus on cardiovascular health. In contrast, during the pandemic, there were significant reductions in hospitalizations for conditions such as NSTEMI and systolic heart failure. This could be a result of patients’ reluctance to seek hospital care because of fear of COVID-19 exposure, potentially leading to underdiagnoses or delayed treatment of these conditions.

In the United States, healthcare spending per capita is the highest globally and has been consistently rising over the past decade [29]. During the COVID-19 pandemic, this trend sharply intensified, with national health expenditures surging by an estimated 9.7% in 2020, a significant leap from the 4.3% increase seen in 2019. This escalation in healthcare costs in the USA can be largely attributed to the high prices of medical labor, pharmaceuticals, and administrative expenses. The index study is a notable reflection of this trend in the cost of hospitalizations for CVDs, which increased by 29.3% in 2020. Contributing factors to this increase include a longer average duration of hospital stays and an increase in the number of hospital procedures over time. Advances in medical technology, while improving treatment outcomes, come with high costs. The price of new cardiovascular drugs, particularly patented medications, significantly increases expenses. An aging population leads to a higher prevalence of CVDs, necessitating more extensive and costly care. In addition, the chronic nature of these diseases requires long-term management, including ongoing medications and possibly repeated hospitalizations, which cumulatively add to healthcare costs. In addition, the pandemic resulted in various other administrative costs, which may have further amplified overall healthcare expenditures.

Study strengths and limitations

The NIS is a comprehensive database for inpatient CVD care, providing detailed insights into sociodemographic factors, hospital costs, and outcomes, representing up to 97% of US hospitalizations. However, there are limitations to this study. The 2020 NIS represents the latest publicly available data, making our analysis largely retrospective. To fully comprehend the pandemic’s impact and determine whether the observed trends persisted beyond our study period, a more extended data analysis is necessary. Furthermore, although we observed associations between the pandemic and various outcomes, such as cardiovascular mortality and hospitalization costs, this does not establish causality. There may be other factors, not considered in this study, that could influence or contribute to these trends.

## Conclusions

Mortality rates from CVDs have risen, particularly in the wake of the COVID-19 pandemic, with significant increases observed among men and minority populations. This increase in mortality has occurred alongside an uptick in hospitalizations for CVDs and an increased use of healthcare resources. There has been a marked increase in the total number of hospital days and procedures, paralleled by a notable surge in the average cost of hospital care for CVD. The primary causes of this increased mortality include myocardial infarction, hypertensive heart disease, heart failure (with or without coexisting chronic kidney disease), unspecified cardiac arrest, atrial fibrillation, nonrheumatic aortic stenosis, and ventricular arrhythmias. Furthermore, mortality rates have recently risen, particularly for conditions such as hypertensive heart disease with chronic kidney disease, hypertensive heart disease with heart failure, ventricular arrhythmias, peripheral angiopathy with gangrene, nonrheumatic mitral valve insufficiency, atrial flutter, orthostatic hypotension, and pericarditis.

This study offers a comprehensive overview of the current trends in hospitalizations and outcomes for patients with CVD, highlighting the impact of recent advancements in diagnostics and care for these conditions, as well as the unique challenges brought forth by the COVID-19 pandemic. There is urgent need for increased surveillance and a concerted effort to enhance diagnosis and treatment for the principal drivers of CVD mortality identified in this study.
